# A case-controlled trial evaluating the summative performance of the 3-D skills Model

**DOI:** 10.1186/s12909-024-05943-9

**Published:** 2024-09-02

**Authors:** C. Robertson, Z. Noonan, J. G. Boyle

**Affiliations:** 1 Undergraduate Medical School, The University of Glasgow, Glasgow, Scotland; 2 Anaesthetics Department, Glasgow Royal Infirmary, 84 Castle Street, Glasgow, G4 0SF Scotland

**Keywords:** Near-peer education, Undergraduate, OSCE, Medical education, Clinical skills

## Abstract

**Background:**

Near-peer teaching is a popular pedagogical teaching tool however many existing models fail to demonstrate benefits in summative OSCE performance. The 3-step deconstructed (3-D)skills near-peer model was recently piloted in undergraduate medicine showing short term improvement in formative OSCE performance utilising social constructivist educational principles. This study aims to assess if 3-D skills model teaching affects summative OSCE grades.

**Methods:**

Seventy-nine third year medical students attended a formative OSCE event at the University of Glasgow receiving an additional 3-minutes per station of either 3-D skills teaching or time-equivalent unguided practice. Students’ summative OSCE results were compared against the year cohort to establish whether there was any difference in time delayed summative OSCE performance.

**Results:**

3-D skills and unguided practice cohorts had comparable demographical data and baseline formative OSCE performance. Both the 3-D skill cohort and unguided practice cohort achieved significantly higher median station pass rates at summative OSCEs than the rest of the year. This correlated to one additional station pass in the 3-D skills cohort, which would increase median grade banding from B to A. The improvement in the unguided practice cohort did not achieve educational significance.

**Conclusion:**

Incorporating the 3-D skills model into a formative OSCE is associated with significantly improved performance at summative OSCEs. This expands on the conflicting literature for formative OSCE sessions which have shown mixed translation to summative performance and suggests merit in institutional investment to improve clinical examination skills.

**Supplementary Information:**

The online version contains supplementary material available at 10.1186/s12909-024-05943-9.

## Background

Near-Peer Teaching (NPT) as an educational tool utilises constructivist principles that can prompt goal orientated learning through constructs such as cognitive congruence [1–3]. Cognitive congruence in the NPT context is described as student tutors and tutees sharing the same knowledge framework [4]. Through this shared understanding, it is proposed that near peer tutors identify the learning needs of tutees easier than content experts [1, 3]. The concept stems from Vygotsky’s work on scaffolded learning where learning is individually calibrated to a learner’s perceived level to effectively solve problems under guidance [5]. Although subject to debate, there is evidence that near-peer tutors can be as effective as experienced faculty if learning adjuncts are used, such as feedback templates or marking sheets [6]. NPT is an attractive complement to undergraduate education with reciprocal benefits received by near-peer tutors and a minimal-cost structure [1–3, 7].

NPT models include formative adaptations of the Objective Structured Clinical Examination (OSCE) [6, 7]. OSCEs are widely used to assess clinical assessment skills including examination skills and procedures. These formative adaptations replicate fidelity and have high student satisfaction rates [7, 8]. Educationally they follow Knowle’s principles of andragogy, supporting experience in driving adult learning [9, 10]. Despite this educational framework, there are conflicting reports of educational attainment with many unable to demonstrate objective performance improvement [7, 8, 11, 12]. Learning models applied to the mock OSCE, such as serial OSCE testing or integrated feedback, aim to achieve objective performance improvements through self-regulated learning principles [7, 8, 12, 13]. Based on Zimmerman’s (2000) self-regulated learning model, a formative OSCE offers a means to assess student’s performance levels, through self-observation of their perceived competence, this can drive self-reflection and further learning [9, 14]. However, undergraduate medical students may be ineffective self-regulated learners and require structured teaching to guide this practice [15–18].

**Fig. 1:**
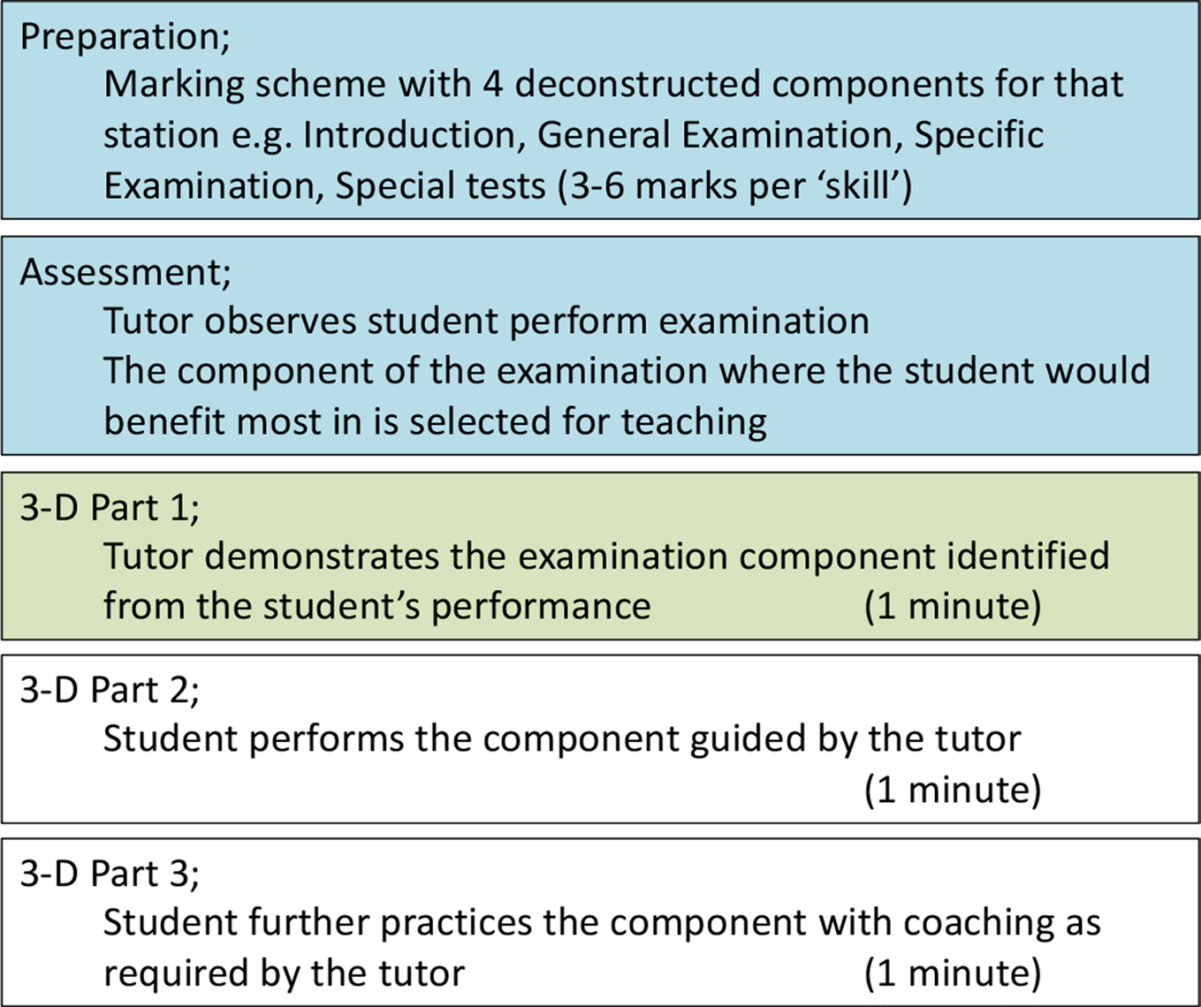
– the 3-D skills model with approximate timings for each composite part

Peyton’s 4-step approach is a well-recognised model for structured psychomotor skill attainment [19]. In Peyton’s model the tutor demonstrates and deconstructs a skill before assessing comprehension and supporting self-practice to reinforce learning [19]. Research would suggest the deconstruction and comprehension stages of the approach constitute the greatest learning gain [19]. Furthermore, whilst an excellent model for initial learning of a psychomotor skill, it requires a significant time investment. This may compromise both Peyton’s models’ practicality in the clinical environment and facility to correct mistakes in existing examination skills. A more focused approach was felt to be advantageous and facilitated our development of the 3-step deconstructed (3-D) skills model(Fig. 1) [18]. Our model integrates the comprehension and self-practice elements of Peyton’s model, structured around a formative OSCE assessment. In our pilot study, we were able to demonstrate comparable student satisfaction and improvements in confidence to other formative OSCE models [7, 8, 18]. We are, however, unsure how the 3-D skills model translates to summative education outcomes, with other models often failing to show such improvement [7, 8]. Our hypothesis is the 3-D skills structured learning model improves subsequent self-regulated learning of psychomotor skills [14]. Our research question was to identify if there was a difference in summative OSCE station pass rates when 3-D skills teaching, or time-equivalent unguided practice, was incorporated into a formative OSCE in comparison to the year cohort.

## Methods

**Fig. 2:**
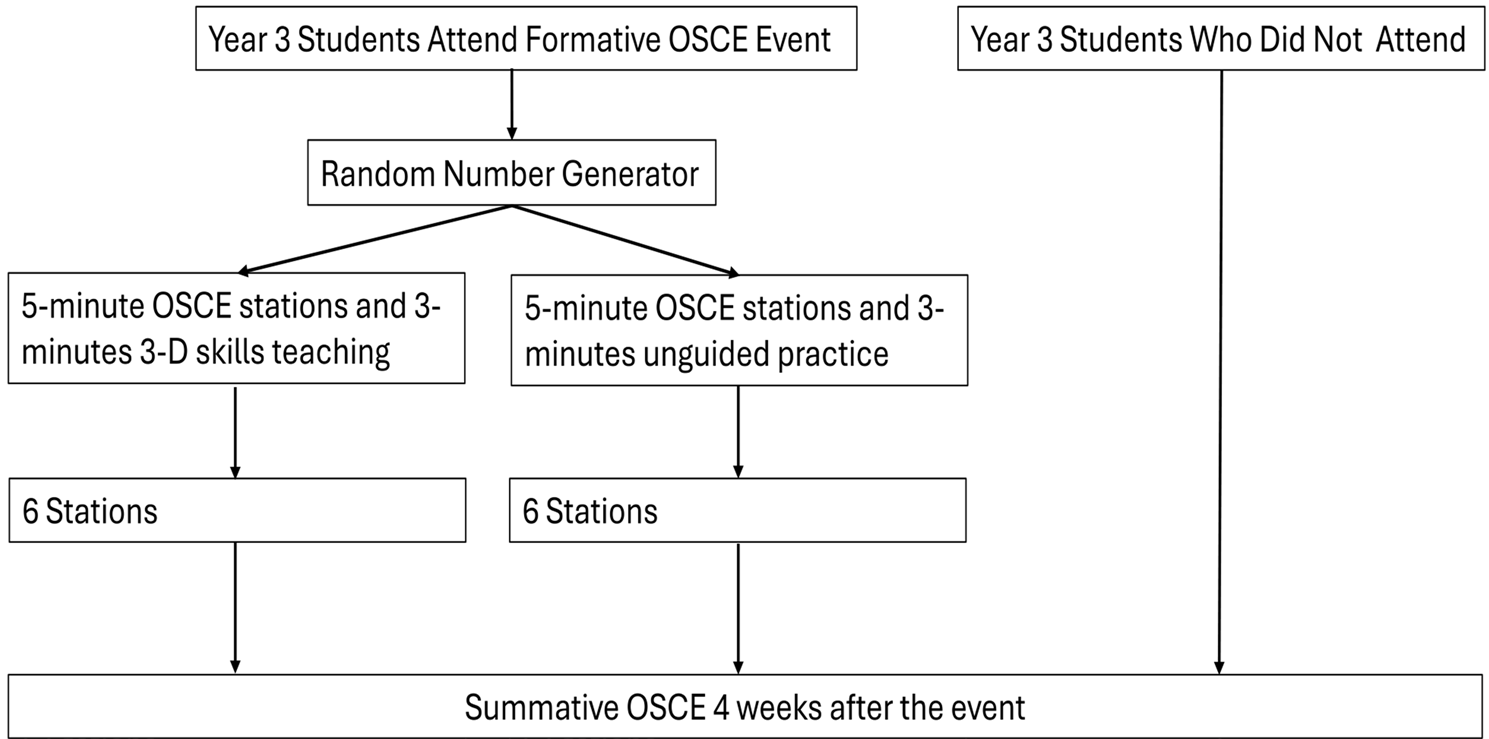
– Study timeline

We designed a case-controlled trial to assess the impact the 3-D skills teaching model has on students’ summative OSCE performance, utilising near-peers as tutors. Students would receive 3-D skills teaching or a time equivalent unguided examination practice after formative OSCE stations, with summative scores compared to the general student population [20](Fig. 2). This allows comparison of attending formative OSCE learning alone to those not attending, and additionally the 3-D skills model to a non-guided comparative control. Our additional comparison enables us to discuss the value of the 3-D skills model in guiding learning over other formative OSCE models such as those utilising unguided examinations only or near-peer led unstructured feedback [7, 11–13].

Student teaching was delivered as a voluntary formative OSCE event using the template from our pilot study. The event ran four weeks prior to the University of Glasgow(UoG) year three summative OSCE examinations [20]. Study approval was granted from the UoG ethics committee. All 278 year three students were invited to participate via campus email and social media with a choice of days and timeslots. Notably these students were on clinical placements during this timeframe. The formative OSCE would be held in the same venue as the upcoming summative OSCE, the clinical skills suite of the University of Glasgow, to maintain fidelity. All work was completed independently from the summative OSCE, and we did not know what stations were likely to appear.

Our pilot study template was adapted in response to student feedback and logistical lessons learned during the event to accommodate an additional examination station as shown in Fig. 2 [20]. The formative event utilised checklist marking sheets sourced from a bank of previous summative OSCEs, each constituting twenty items arranged in a binary (done/not done) scoring metric with a global assessment category of pass, borderline pass or fail. The marking sheets were domain-based, aligned with summative OSCE marking processes at Glasgow. Our circuit composed of six examination stations: abdominal, cardiovascular, upper limb neurological, knee joint, hip joint and respiratory. For background, students are formally taught the corresponding clinical skills between years 2 and 3 by experienced faculty, often using Peyton’s 4-step approach. Additionally, we replicated the assessment of our pilot study’s short-term attainment of the 3-D skills model with the larger cohort in this study, further details and results can be found in supplementary material 1. Fidelity was monitored by faculty to observe compliance and enforce examination conditions, for example adherence to station timings. At the end of the event, all students were informed of their checklist scores for each station.

On enrolment to the study, students were allocated to a date, timeslot and either 3-minutes of 3-D skills teaching or 3-minutes of unguided practice groups via a random number generator. Both cohorts were instructed to attend separate rooms where a member of the faculty would take attendance, consent and provide an identical pre-session briefing to students. This brief stated that “each station will comprise a five-minute formative OSCE station and three-minutes of practice to go over any part of the examination. These three-minutes of practice time may or may not be guided by one of the examiners.” There was no crossover between student cohorts, and they were not informed which cohort they were allocated to. The specific difference between the cohorts is the 3-minutes after each OSCE station in whether it is guided (3-D skills) or unguided.

Student tutors in their 4th year or above were recruited to teach at the formative event. These tutors were from the Clinical Assessment Practice (CAP) Glasgow student society which regularly runs formative OSCE events for Glasgow students. These tutors attended a half day training event run by a team of junior doctors, experienced with the 3-D skills model, with the instructional design replicated from our pilot study [9]. Tutors alternated as simulated patient or tutor for both cohorts and attended separate cohort briefings [9]. As per the pilot, external markers were recruited from foundation year two and above to formally mark third-year students [9]. These external markers were not briefed on the differences between cohorts with no event circuit cross-over [9].

## Data collection

To power our study’s summative analysis, we analysed data from the previous year three summative examination results and determined that the median station pass rate was 11/12. Using a 2-point continuous study power calculation we estimated that we would need a total sample size of 58 students (29 per study arm) with 1:1 enrolment, achieving an alpha value of 0.05 and power of 0.8 to detect a difference of 1 in summative station pass rates. Our rationale was OSCE grade banding is linked to overall number of stations passed, therefore an additional station pass is likely to affect grading and thus correlate to educational significance. We sourced summative results from both cohorts and year 3 students who did not attend the event.

We used the IBM SPSS Statistics Software (version 27) to process our data. Our data set suggested that the summative stations passed exhibited a left skewed distribution. We listed the sample size, range, median and mean rank score for summative descriptive statistics. To compare the 3 populations, 3-D skills, unguided practice, and general population, we used the Kruskal-Wallis-test with a *p* < 0.05 correlating to statistical significance, and the null hypothesis that there are no significant differences between the groups. Multiple comparisons using the Post-hoc Dunn-Bonferroni test were made to identify statistically significant differences between the 3 populations, a Bonferroni corrected alpha of 0.017 indicated statistical significance.

Demographical data was obtained from a pre-event questionnaire that was filled in immediately preceding the event (Table 1). All other formative assessment results including event satisfaction, confidence levels and checklist scores from 1st -2nd sitting are described in supplementary material 1.

## Results

### Demographics

In total 96 students expressed interest to attend our formative course (35% of year 3). Two students in the unguided practice cohort did not consent for use of their data. Of the remaining 94 students, 13 were unable to attend at short notice, citing competing educational events. In summary, 46 students completed the 3-D skills teaching and 33 completed the unguided practice. Demographics for the two cohorts and all of the year 3 cohort are shown in Table 1.

**Table 1:** – unguided practice, 3-D skills and all the year 3 cohort demographics

	3-D Skills (*n* = 46)	Unguided practice (*n* = 33)	All of year 3 (*n* = 278)
Male	16 (35%)	10 (30%)	39%
Female	30 (65%)	23 (70%)	61%
Non-U.K.	20 (43%)	11 (33%)	14.5%
U.K.	26 (57%)	22 (67%)	85.5%
18–22	34 (74%)	24 (73%)	77.6%
23 or older	12 (26%)	9 (27%)	22.4%

Our demographics approximate to the year 3 averages for gender and age, however, we have a disproportionately high percentage of non-U.K. students included in the study, a demographic feature that was also noted in the pilot [9].

### Summative student results

Each student sat 12 OSCE stations in the summative examination. Descriptive statistics can be found in Table 2.

**Table 2:** Summative OSCE descriptive statistics

	3-D Skills Cohort (*n* = 46)	Unguided Practice Cohort *n* = 33)	Year 3 students who did not attend the formative event (*n* = 199)
Range Stations Passed	10–12	8–12	7–12
Median Stations Passed	12	11	11
Mean Rank Score	202	173	119

The Kruskal-Wallis-test rejected the null hypothesis, confirming a statistically significant difference in the dependent variable (stations passed) between the three groups (*P* < 0.01). The Post-Hoc Dunn’s test using the Bonferroni corrected alpha of 0.017 indicated that the mean ranks comparing the 3-D skills cohort to the other year 3 students and the unguided practice cohort to the other year 3 students were significant(*P* < 0.01). The mean rank difference of the 3-D skills cohort to the unguided practice cohort did not reach significance (*p* = 0.099).

### Grade correlation

Summative examination results correlated an A grade to students achieving 12/12 stations passed, a B grade to students achieving 11/12 and C for those achieving 10/12 passed stations. This suggests the differences between median grade for the 3-D skills cohort (12/12 stations passed) and the rest of year 3 (11/12 stations passed) would be both statistically and educationally significant. However, as the unguided practice cohort and the year 3 students who did not attend achieved the same median pass rate (11/12 stations passed), although statistically significant this result did not reach educational significance.

## Discussion

Students who attended the formative OSCE event had a significantly higher summative grade than those who did not attend, regardless of if they were in the 3-D skills or unguided practice cohort. Whilst reaching statistical significance, the unguided practice cohort achieved the same median grade than students not attending the event, questioning the educational significance. In contrast, the 3-D skills cohort achieved a median higher-grade banding, correlating to an A grade, suggesting educational significance. Nevertheless, both cohorts demonstrate the value of near-peer learning in curricula and achieved impact at subsequent summative OSCE examinations.

With minimal training, near-peer tutors were able to utilise the 3-D skills model as a teaching aid, achieving a positive impact on summative examination performance. This supports previous evidence that near-peer tutors can be as effective as faculty [6, 20]. Many Universities now offer support for prospective near-peer tutors and our study provides further validation. Sustainability is evidenced by the large pool of voluntary tutors we recruited through altruistic intent to teach. Our half-day student tutor training session demonstrates a popular formative model, being faculty and resource light, offering summative assessment translation and synergistic tutor benefits. We believe this warrants further investment to promote near-peer education [2, 3, 8, 20].

Assessing generalisability, we initially recruited 35% of year 3 students to attend the formative OSCE event. Given the ‘out-of-hours’ format with voluntary attendance, we considered this a reasonable response. Whilst effort was taken for the event to be accessible to all, offering a range of dates and timeslots, we may be inadvertently disadvantaging some student cohorts through an out-of-hours model. Although no students approached us for alternative arrangements, we can argue with the now demonstrated improvement at time-delayed summative examinations, we should incorporate the 3-D skills formative assessment into our main curricula to ensure no student is disadvantaged. Gender mix was approximate to the year average, suggesting gender would be unlikely to contribute to the results [21–25]. A notable trend in this study, and our pilot, is a disproportionately high rate of non-U.K. attendees [20]. We had approximately twice as many non-U.K.students across both cohorts than we would proportionally expect. Studies suggest overseas students perform less well in OSCEs than home/native students, and an awareness of this, coupled with a heightened conscientiousness in this group may be responsible for the higher attendance rates [24, 26, 27]. The nature of this is unclear with language and cultural constructs postulated as discerning factors [24, 26, 27]. With the popularity of NPT, endeavours such as the 3-D skills model may offer an ideal solution to bridge the perceived educational gap and afford overseas students’ equal opportunities for success at summative OSCE assessments as home/native students.

Although the comparison of median rank scores between the summative 3-D skills and unguided practice cohort did not reach significance, we can argue this arm was underpowered to detect median differences in cohorts less than 1 station. We observed a significant drop in attending student numbers on the night, affecting the unguided practice cohort disproportionately. This was due to a competing University event, highlighting the challenges and limitations experienced with out-of-hours vocational education. Whilst we did not demonstrate statistical significance between the two cohorts, we can argue the superiority of the 3-D skills approach based on current literature. Research suggests junior medical students may have underdeveloped self-regulated learning skills and thus require mentorship to reach efficacy [17, 18]. Through this extension, perhaps subsequent self-regulated learning facilitated by the 3-D skills mentored approach was the driving factor for summative success. Nevertheless, achieving a statistically significant increased summative grade through facilitated OSCE learning, guided-or-unguided, expands on the conflicting literature on medium term impact of near-peer formative OSCE models [7, 8, 12]. Reaching educational significance by increasing students’ grade banding furthers the argument to utilise the 3-D skills model in formative OSCE teaching.

Our hypothesis for the 3-D skills models’ effectiveness focuses on constructivist theory, supporting scaffolded learning with cognitive congruity of near-peer tutors [1, 4, 5]. Utilising the 3-D skills model provides a structure to help near-peer tutors engage effectively with learners utilising their own experiences. Our hypothesis echoes the premiss of Loda et al [5] in their discussion surrounding the importance of cognitive and social congruence in establishing effective NPT sessions, originating from Schmidt and Moust’s model [28]. The adaptations to teaching technique and perception of task difficulty is likely instrumental in creating realistic learning outcomes from NPT [17]. In future work we seek to demonstrate the educational utility of the 3-D skills model on clinical placements. We suggest the 3-D skills model may be effectively paired with formative tools such as the ‘Mini-Clinical Evaluation Exercise’ offering a means of assessing learners prior to 3-D part 1(Fig. 1) [29].

A limitation in interpreting our results is student baseline performance. We did not have access to any previously captured descriptor metrics, such as written summative performances which may have further described student baseline. We can also argue hermeneutically that higher performing students are likely to attend additional teaching events, suggesting bias. Current research would disprove this notion however, as conscientiousness alone does not appear to influence examination results [30, 31]. Furthermore, previous formative OSCE only models have failed to demonstrate statistically significant improvements from baseline student performance in summative assessments [7, 8]. Perhaps subsequent self-regulated learning driven by the 3-minutes of guided-or-unguided teaching is responsible for the differences in summative grade.

An additional limitation was the difficulty in performing longitudinal follow up of this cohort due to the COVID-19 pandemic. Indeed, this cohort were the first to sit a ‘VOSCE’ in later years, a rapidly implemented novel adaptation to the OSCE program using a virtual interface [32]. However, only a small proportion of students completed this novel approach, preventing meaningful comparison.

As demonstrated in supplementary material 1, the short-term impact of the 3-D skills model has been replicated with a larger sample size. This model achieves high student satisfaction, improvements in confidence, a mean increase of ~ 15% checklist scores and a significantly higher-grade banding than a time-controlled unguided equivalent.

In future work we aim to incorporate this model into our curriculum, offering all students access to a formative OSCE with additional guided teaching. We also wish to test if the 3-D skills model can be readily applied to other formative assessment tools such as the supervised learning activities completed on clinical placements, e.g., Mini-CEX. If we can demonstrate further expansion on quantitative and qualitative outcomes of the 3-D skills model in clinical education, we may encourage other centres to adapt this model to their own curricula.

## Conclusion

We have demonstrated that student engagement with a formative OSCE incorporating guided-or-unguided practice can statistically increase students’ performance at summative OSCEs. The 3-D skills teaching model (guided practice) achieved educational significance by demonstrably improving student median grade banding in subsequent summative OSCE performance, when compared with students receiving unguided OSCE practice. Our data validates the importance of guided teaching to support further self-regulated learning in novice learners. Future work will focus on curriculum integration both in-and-out of clinical placements to demonstrate the adaptability of the 3-D skills model.

### Electronic supplementary material

Below is the link to the electronic supplementary material.

Supplementary Material 1

Supplementary Material 2

## Data Availability

All data generated or analysed during this study are included in the published article [and its supplementary information files].

## Abbreviations

3: D skills model–3–step deconstructed skills model
CAP: Clinical Assessment Practice
COVID: 19–coronavirus disease 2019
NPT: Near–Peer Teaching
OSCE: Objective Structured Clinical Examination
UoG: University of Glasgow
VOSCE: Virtual Objective Structured Clinical Examination
