# Supplementary material for: A case-controlled trial evaluating the summative performance of the 3-D skills Model
Source: BMC Med Educ. 2024 Sep 2;24:954. doi: 10.1186/s12909-024-05943-9 (PMC11370037; doi:10.1186/s12909-024-05943-9)
Supplement: Supplementary file 2 — Supplementary Material 2 [file 12909_2024_5943_MOESM2_ESM.docx]

# Formative Event Results

We replicated the 3D skills pilot study assessment to determine if comparable student satisfaction and improvements in student confidence would be demonstrated with the 79 students who attended the event [20]. This was included as part of our University of Glasgow ethics committee application and approved by the school of life sciences.

In comparison to the pilot we assessed the abdominal examination in station 1 and reassessed it after students had completed the other 5 stations in their circuit.

## Data Collection:

External markers (Foundation doctor year 2 and above), collected checklist scoring sheets for station 1 on first sitting and on its repeat after students had sat the other 5 stations in the formative OSCE circuit.

Students were asked to complete pre-and-post event questionnaires which contained anonymised student numbers to link data. These questions included self-identified age group, gender, ethnicity and 5-point Likert scales for confidence to sit a summative OSCE and event satisfaction.

## Data analysis

***Ordinal data***

Student pre-and-post confidence results were described using the median and Wilcoxon Signed Rank Test, with a p value<0.05 correlating to statistical significance. Baseline confidence levels would be compared using the Mann Whitney U test.

Student satisfaction levels were described using median for descriptive statistics and the Mann Whitney U test to assess statistical significance.

**Continuous data**

Checklist scores from 1^st^ to 2^nd^ sitting were assessed using sample mean, standard deviation, and paired student t-testing, with p<0.05 correlating to significance. To assess baseline performance, 1^st^ checklist scores would be compared using the unpaired student t-test.

**Global Assessment Rating**

Global assessment rating (pass, borderline pass, fail) as evaluated by the external examiners was evaluated using the borderline regression method to establish a station pass mark. Baseline 1^st^ sitting performance and 1^st^-2^nd^ sitting pass rate would be compared statistically using the Mann Whitney U test, with p<0.05 correlating to significance.

**Fidelity assessment**

All student tutors and external markers were debriefed independently at the end of the session to confirm both cohorts adhered to the brief. Any concerns were summarised and anecdotally described.

## Results

**Ordinal Data**

|  | Median event satisfaction | Median confidence to sit the summative OSCE (pre-event) | Median confidence to sit the summative OSCE (post-event) |
| --- | --- | --- | --- |
| Unguided practice cohort | 5/5 | 3/5 | 4/5 |
| 3-D skills cohort | 5/5 | 3/5 | 4/5 |

Event satisfaction was not statistically different between the two cohorts (U test, p=0.27). Both cohorts had a significant increase in median confidence from 3/5 to 4/5 (Signed Rank test, p<0.01). The difference in baseline confidence was non-significant (U test, p=0.69).

**Continuous Data**

|  | Mean 1^st^ sitting score & Standard deviation | Mean 2^nd^ sitting score & Standard deviation |
| --- | --- | --- |
| Unguided practice cohort | 15.5/20 (2.69) | 16.0/20 (2.98) |
| 3-D skills cohort | 14.5/20 (2.44) | 17.8/20 (1.89) |

The 3-D skills cohort had a significant increase of 3.3 checklist marks from 1^st^ to 2^nd^ sitting (t test, p<0.01). The unguided practice cohort also demonstrated a significant improvement of 0.5 marks (t test, p=0.03). Comparing the baseline performance of both cohorts there was a difference of 1 checklist mark which was found to be non-significant on student t test (p=0.09). Given the close approximation to a significant result and the difference in sample size we repeated the baseline performance calculation with the Mann Whitney U test which was also non-significant (p=0.07).

## Baseline performance at formative OSCE

The unguided practice group achieved a median checklist score of 15.5 on their first sitting of station 1 compared to 15 with the 3-D skills group. This was found to be non-significant (U test, p=0.09).

**Global assessment rating**

1=Fail, 2=Borderline, 3=Pass

From the Borderline Regression Method, pass mark set at 12.4/20.

During the first sitting this corresponds to 37/46 students passing in the 3-D skills cohort and 30/33 students passing in the control cohort.

During the second sitting all 46/46 students in the 3-D skills cohort’s checklist score would have passed the 12.5 benchmark, whereas 28/33 students would have passed in the control.

In the 3-D skills group, the pass rate was significantly higher from 1^st^-2^nd^ sitting (p<0.01). Comparing the unguided practice cohort’s 1^st^-2^nd^ sitting pass rate was found to be non-significant (p=0.46). The baseline pass-rate of both cohorts was found to be non-significant (p=0.21).

**Fidelity Assessment**

There were no detected breaches from study protocol by the student-tutors. Tutors in the unguided practice cohort did express frustration being unable to offer feedback to students during the 3-minutes, suggesting some students used this time ineffectively. Some tutors in the 3-D skills cohort suggested it was difficult to pick one area to focus teaching on in weaker student performances.
